# COLOSTOMY CLOSURE: RISK FACTORS FOR COMPLICATIONS

**DOI:** 10.1590/0102-6720201700040001

**Published:** 2017

**Authors:** Alexandre Z. FONSECA, Edson URAMOTO, Otto M. SANTOS-ROSA, Stephanie SANTIN, Marcelo RIBEIRO-JR

**Affiliations:** 1 Department of Surgery, University of Santo Amaro, São Paulo, SP, Brazil.

**Keywords:** Colostomy, Surgical procedures, Postoperative, Complications., Colostomia, Procedimentos cirúrgicos, Complicações pós-operatórias

## Abstract

***Background*:**

*:* The restoration of intestinal continuity is an elective procedure that is not free of complications; on the contrary, many studies have proven a high level of morbidity and mortality. It is multifactorial, and has factors inherent to the patients and to the surgical technique.

***Aim*:**

*:* To identify epidemiological features of patients that underwent ostomy closure analyzing the information about the surgical procedure and its arising complications.

***Method*:**

*:* It was realized a retrospective analysis of medical records of patients who underwent ostomy closure over a period of seven years (2009-2015).

***Results*:**

*:* A total of 39 patients were included, 53.8% male and 46.2% female, with mean age of 52.4 years. Hartmann´s procedure and ileostomy were the mainly reasons for restoration of intestinal continuity, representing together 87%. Termino-terminal anastomosis was performed in 71.8% of cases, by using mainly the manual technique. 25.6% developed complications, highlighting anastomotic leakage; there were three deaths (7.6%). The surgical time, the necessity of ICU and blood transfusion significantly related to post-operative complications.

***Conclusion*:**

*:* It was found that the majority of the patients were male, with an average age of 52 years. It was observed that the surgical time, the necessity of blood transfusion and ICU were factors significantly associated with complications.

## RESUMO

***Racional*:**

A reconstrução de trânsito intestinal é procedimento realizado eletivamente que não é isento de complicações, pelo contrário, muitos estudos evidenciam alto grau de morbimortalidade, dependendo de fatores inerentes ao paciente, bem como da própria técnica operatória.

***Objetivo*:**

Identificar as características epidemiológicas dos pacientes submetidos à reconstrução intestinal, além de analisar as informações a respeito do procedimento cirúrgico e as complicações decorrentes.

***Método*:**

Foi realizado análise retrospectiva dos prontuários dos pacientes submetidos à reconstrução intestinal no período de sete anos (2009-2015).

***Resultado*:**

Foram incluídos 39 pacientes, sendo 53,8% homens e 46,2% mulheres, com idade média de 52 anos. As operações tipo Hartmann e ileostomia foram os motivos para a reconstrução do trânsito intestinal, representando juntas 87% dos pacientes. A anastomose terminoterminal foi realizada em 71,8% dos casos, utilizando principalmente a técnica manual. 25,6% dos pacientes apresentaram complicações, destacando-se a fístula de anastomose. Três (7,6%) morreram. O tempo operatório, necessidade de UTI e transfusão sanguínea apresentaram significância estatística com as complicações pós-operatórias.

***Conclusão*:**

Verificou-se que a maioria foi de homens, com média de idade de 52 anos. Entre as variáveis estudadas, observou-se que a duração da operação, a necessidade de transfusão sanguínea e de UTI foram fatores complicadores com significância estatística.

## INTRODUCTION

Ostomy creation is used mainly for fecal diversion as a treatment option for colonic diseases^20^. All segments of the colon can be used, as well as the distal part of the ileum. Hartmann’s procedure was first described in the early 1920’s by the French surgeon that named the procedure and was initially performed in patients with left colon neoplastic obstruction; the intention was to decrease mortality due to anastomotic leakage^23^. With time, its indication has been extended to benign disorders such as complicated diverticulitis, gunshot wound to the colon and complications after primary colonic anastomosis. It is usually performed in the emergency setting when primary anastomosis is unfeasible, especially in haemodynamically unstable patients due to sepsis and multiorgan dysfunction^11^. Currently, when resection of the left colon is needed, a single stage procedure with primary anastomosis is preferred by most surgeons^8^ ^,^ ^7^. 

Several techniques of intestinal continuity restoration have been described over the past decades. Stomas are usually temporary but in up to 74% of the cases they become permanent; this is owed to several and different factors, such as age, rectal stump size and patient’s comorbidities^29^ ^,^ ^10^. Loop ileostomies and sigmoid colostomies have significant higher rates of reversal, being the former five times more likely to be reversed^10^.

Restoring intestinal continuity can be a challenging procedure and many factors are involved in its timing. The attending physician should consider it as complex surgery^8^ ^,^ ^9^. Besides that, patients have a high risk of developing complications due to their comorbidities and prior surgery; thus, careful patient selection is essential^13^. Complication rates as high as almost 55% and mortality up to 4% have been described^23^ ^,^ ^5^ ^,^ ^25^ ^,^ ^3^ ^,^ ^4^. Anticipating and identifying complications are essential and could lead to better outcomes.

The aim of this study was to identify factors associated with morbidity in patients submitted to restoration of intestinal continuity for a variety of diseases. 

## METHODS

Data on consecutive patients submitted to restoration of intestinal continuity between January 2012 and October 2015 were retrospectively analyzed. This included patient demographic characteristics, indication for stoma creation, details of the surgical treatment, type of procedure, type and method of anastomosis, postoperative adverse events, length of hospital stay, surgical time, abdominal drainage, need of intensive care unit (ICU), blood transfusion, associated procedures, American Society of Anesthesiology (ASA) score, morbidity and mortality.

The main end point was to establish a significant relationship between these variables and the incidence of complications. Mortality was defined as death occurring in the first 30 postoperative days; morbidity was defined as any type of complication that lead to increase in hospital stay or the need of medical intervention (examples: drainage and reoperation). They consisted in adverse events related to the procedure, such as postoperative ileus, anastomotic leakage, wound infection, bowel obstruction and evisceration. Anastomotic leakage was defined as: anastomotic dehiscence leading to peritonitis, presence of fecal content the in pelvic drain and the presence of an abscess surrounding the anastomosis with oral or rectal contrast leakage seen at CT. Not all patients with leakages were submitted to a new procedure; only those with generalized peritonitis, sepsis or multiorgan dysfunction were re-operated. Wound infection was defined as skin erythema, increase in temperature or fluid with positive culture requiring antibiotics or local intervention.

All surgeries were performed via laparotomy through a midline incision and under general anesthesia; they were all performed by the same medical staff. Mechanic bowel preparation was not used; all patients were asked to keep an oral liquid diet for five days prior to surgery. All rectal stumps were assessed by barium enema and the remaining of the colon screened by colonoscopy. No protective colostomy or ileostomy was performed in this series. 

Postoperative ileus was defined as prolonged recovery of bowel function with the need of reinsertion of a nasogastric tube increasing hospital stay. Preoperative antibiotics were given in all cases with therapeutic purposes; different regimens were utilized. 

Anastomosis were either handsewn or stapled. When manually performed, a double layer of uninterrupted absorbable suture was used. When stapled, a circular stapler was used (CDH 33A Ethicon®) to manufacture an end-to-end anastomosis; when the side-to-side technique was preferred, a linear cutter (TLC75 Ethicon®) was used. The stoma was released from the skin and after mobilization, the anvil was introduced with a purse string suture; after adhelyosis, the rectal stump was identified and anastomosis was performed. Abdominal drainage was used at the surgeon’s discretion.

Blood requirement during the procedure was based on intraoperative blood loss that lead to hemodynamic repercussion and hemoglobin level <10 g/dl. 

ICU requirement was based on the patient’s age (>65 years), prolonged surgical time (no time limit was defined and this decision was made in conjunction between the surgical team and anesthesiologists) and postoperative hemodynamic instability. 

### Statistical analysis

Was performed using the Student’s t test, Fisher exact test and chi-squared test (x^2^). Significance level was defined as p< 0.05. 

## RESULTS

Thirty-nine patients were submitted to reversal of Hartmann’s procedure; 21 (53.8%) were men and 18 (46.2%) women. The mean age was 52.48 years (14-77). ASA grades were as follow: 25 (64.1%) patients were ASA I and 14 (35.9%) were ASA II; no ASA III and IV patients were submitted to the procedure. Different ASA grades were not significant risk factors for complications (p=0.55). 

All patients were initially submitted to a Hartamnn’s procedure and consequently had left sided end colostomies. Patients’ index stoma indications are summarized in Table 1. Index indication was not a significant risk factor for morbidity (p=0.53). 

**TABLE 1:** Stoma index indication and the incidence of complications

	Complication		p= 0.5360
	Yes n (%)	No n (%)	
Colon malignancy	2 (20)	5 (16.1)	
Diverticulitis	1 (10)	4 (12.9)	
Megacolon	1 (10)	4 (12.9)	
Gunshot wound	1 (10)	3 (9.6)	
Stab wound	1 (10)	0	
Unknown	1 (10)	4(12.9)	
Other	1 (10)	11 (35.4)	
Total	8 (100)	31 (100)	

Complications were encountered in eight patients (20.5%). The most common adverse event was anastomotic leakage; complications are reported in Table 2. Reoperation was necessary in seven of eight subjects. From these seven, four patients survived. One of them needed reoperation for abdominal wall closure due to evisceration; another was submitted to fistula closure and protective ileostomy; a third needed reoperation for fistula closure and abdominal drainage; in the fourth, creation of a new stoma was necessary due to anastomotic leakage. The need of reoperation was not a significant risk factor for morbidity (p=0.47). 

**TABLE 2:** Complications on restoration of transit continuity

Complication	Death n (%)	Discharge n (%)	Total n (%)
Leakage	1 (33.3)	2 (40)	3 (37.5)
Leakage and evisceration	0	1 (20)	1 (10)
Bowel obstruction	1 (33.3)	0	1 (10)
Evisceration	0	1 (20)	1 (10)
Wound infection	0	1 (20)	1 (10)
Reconstruction unfeasibility	1 (33.3)	0	1 (10)
Total	3 (100)	5 (100)	8 (100)

Three patients died and all of them were submitted to a second procedure; one of them had an anastomotic leak, being submitted to a new laparotomy with fistula closure and protective ileostomy and negative pressure wound therapy; he died from septic complications and multiorgan dysfunction. The second patient also died from infectious complications and multiorgan dysfunction due to missed small bowel injury. Finally, in the last patient, stoma closure was not feasible due to technical reasons. In his reoperation, a missed small bowel injury was found. He was critically ill and was submitted to negative pressure wound therapy; he died from pulmonary complications. Patients who had complications had significantly more chances of dying (p*=*0.01).

 End-to-end anastomosis was the preferred configuration (71.8%), followed by side-to-side (15.3%) and end-to-side (12.8%); anastomosis configuration was not significantly associated with adverse events (p=0.82). Handsewn anastomosis was performed in 56.5%. Type of anastomosis was also not significantly associated with complications (p=0.46). 

Overall hospital stay ranged from 4-41 days (mean 11.9); mean overall surgical time was 211.2 min (90-550). Patients with complications had significant longer mean surgical time (p=0.02; 240x165 min). Likewise and as expected, patients with adverse events had significant longer hospital stay (p*=* 0.0002; 24x8 days). 

Blood transfusion was necessary in five patients and ICU stay after reversal in eight. These two factors were also significantly associated with complications (p*=*0.01 and p=0.001 respectively). 

Abdominal drainage was performed in 22 (56.41%) cases; of these, four had anastomotic dehiscence. Two of them needed reoperation; thus, due to the low number of this specific type of patient, we could not evaluate the effectiveness of abdominal drainage in preventing the need of new interventions. The presence or absence of drains was not significantly linked with complications (p*=*0.28). 

Eleven associated procedures were performed. They are summarized in Table 3. This factor was not associated with complications (p*=*0.53). 

**TABLE 3:** Associated procedures

Procedure	n (%)
Incisional herniorraphy	5 (45.4)
Enterectomy	3 (27.2)
Appendidectomy	1 (9)
Colectomy	1 (9)
Sigmoidectomy	1 (9)
Total	11 (100)

## DISCUSSION

Currently, primary anastomosis is preferred by most surgeons, with good outcomes^8^ ^,^ ^7^ ^,^ ^17^ ^,^ ^6^. One of the reasons for this option is the high morbidity when stoma closure is performed. Complication rates over 50% have been described, with a high 4% mortality rate^23^ ^,^ ^13^ ^,^ ^5^ ^,^ ^25^ ^,^ ^3^ ^,^ ^4^ ^,^ ^15^. Our complication rate of 20.5% is similar to several studies^13^ ^,^ ^12^. This can be explained by patient’s advanced age, comorbidities and the complex index surgery that lead to stoma creation; its closure should be considered as difficult and challenging as the initial intervention. With this in mind, it is expected that not all subjects are candidates to reversal. Proper patient selection is essential for good outcomes. 

In this matter, Hodgson *et al.* retrospectively analyzed their data on 165 patients discharged after Hartmann’s procedure and studied their reversal rates and patient parameters associated with reversal^15^. They found out that the procedure was feasible in 45% of the subjects. Patients under 70 years with longer interval from their index surgery (6 and 12 months) were more likely to get reversal. Age, comorbidities and patient refusal were the main reasons for non-reversal. An interesting point discussed by the authors was that the majority of the patients who decided not to undergo reversal had been in the ICU; they suggest that this stay after surgery could lead to post-traumatic stress disorder and discourage them to closure their stoma. Adequate treatment could increase reversal rates. Salem *et al.* published similar results^24^. In their study, patients under 50 years of age were reversed in more than 80% of the cases and those with over 70 years were reversed in less than 30%. 

Timing for stoma closure remains in debate. There are two mainstreams: those who believe that early reconstruction is better and those who prefer to operate on patients after a longer period of time from the index surgery. The latter group believes the optimal time is after total resolution of the inflammatory scenario in the patient’s abdomen and with better nutrition. Shorter time to reversal would possibly avoid rectal stump atrophy. Nevertheless, some groups could not find any difference in reversal and complication rates in both time period^4^ ^,^ ^6^ ^,^ ^12^.

As expected, in this study, for patients who developed complications, hospital stay and ICU stay were significantly longer, blood transfusion was more required and they had significant longer surgical time. This has also been described by other authors^9^ ^,^ ^25^ ^,^ ^3^ ^,^ ^15^ ^,^ ^3^ ^,^ ^16^. Keller *et al.* suggested in their paper that patients identified with these factors should have different discharge planning to avoid readmissions^22^. The relationship between blood requirement and complications is not described in the literature. The possible rationale for this is that with less circulating blood, blood supply to the anastomosis decreases leading to local complications, such as leakage. In our opinion, patients in shock are still one of the few indications for stoma creation (regardless the degree of peritoneal contamination), although other strategies such as primary anastomosis with diverting stoma can be performed. Specifically, blood transfusion has been associated with infections^2^ ^,^ ^3^. Immunosuppression caused by transfusion is believed to mediate these infections. 

Age has been considered by some to significantly increase the incidence of complications^6^ ^,^ ^22^. Nevertheless, it was not a risk factor in the current study; this is in accordance with data of other authors^25^. Usually older patients owe their stomas to cancer and this state leads to health deterioration, which would lead to more complications. Younger patients tend to be healthier at baseline and usually have their stomas created due to trauma and are expected to have less complications. Despite this, we did not encounter more complications occurring in subjects with neoplasms. 

Another interesting topic is whether the configuration and type of anastomosis would directly interfere in surgical complications especially in training centers. In the present study, these variables were not associated with complications as corroborated by other studies^25^ ^,^ ^18^ ^,^ ^21^. Aydin *et al.* found different results^4^. Handsewn anastomosis was a significant risk factor for leakage in their paper; however, there was a selection bias, where these patients had more peritoneal contamination. Roig *et al.* also shared similar results^22^. Leakage was significantly more common in handsewn and side-to-side anastomosis. 

One factor that could possibly decrease the incidence of complications is performing stoma reversal laparoscopically^28^. This approach leads to fewer complications, less blood loss (related with complications in the present study), shorter hospital stay and faster return of bowel movements^25^ ^,^ ^26^ ^,^ ^19^. 

Avoiding stoma creation would be the best option. Surgeons have used alternative techniques in the management of colonic diseases in the acute setting. Primary anastomosis with diverting loop ileostomy or colostomy is technically feasible. This technique in association with pelvic drainage is also used in the management of anastomotic leaks^7^. Diverting loop diverts fecal content and drainage leads to adequate sepsis control; this avoids dismantling the anastomosis or managing it in an inflammatory field. A recent meta-analysis investigating complications of diverting loop stomas found out that closure of loop ileostomies compared to loop colostomies had significantly fewer wound infections and incisional hernias; overall complications were not different^14^. Sier *et al.* published their results and concluded that loop ileostomies were 4.3 more likely to be reversed than end ileostomies^27^. Based on these findings, the type of ostomy should be carefully selected in the index surgery. Closure of loop ileostomy is believed to have fewer complications than loop colostomy^10^ ^,^ ^13^. 

Another option is laparoscopic peritoneal lavage with drainage. A recent randomized trial showed interesting and promising results in Hinchey III patients when compared to Hartmann’s procedure^1^. There was no statistical difference in reoperation rates, complications and blood transfusion; those submitted to laparoscopic lavage had shorter operating time and hospital stay. As the authors mentioned, this may have clinical implications on the management of complicated diverticulitis. 

## CONCLUSION

Morbidity and mortality following stoma closure are not insignificant so proper patient selection is essential. Blood transfusion, ICU stay and longer operating time were significantly associated with complications. Patients identified with these specific factors should have different discharge planning to avoid unnecessary readmissions. 
